# Experiences and Expectations of Immigrant and Nonimmigrant Older Adults Regarding eHealth Services: Qualitative Interview Study

**DOI:** 10.2196/64249

**Published:** 2025-03-14

**Authors:** Maedeh Ghorbanian Zolbin, Sari Kujala, Isto Huvila

**Affiliations:** 1 Department of Computer Science Aalto University Espoo Finland; 2 Department of ALM Uppsala University Uppsala Sweden

**Keywords:** eHealth services, older adults, immigrant, usability, user experience, emotion, self-determination theory

## Abstract

**Background:**

The emergence of eHealth services could contribute to improving individuals’ quality of life by optimizing effective and efficient care. However, various challenges might limit some older adults’ use of eHealth services.

**Objective:**

This study aimed to understand the perspectives of older adults (aged ≥65 years) of different backgrounds regarding eHealth services. We explored the experiences of Iranian immigrant and nonimmigrant older adults with eHealth services to identify their perceived challenges, emotions, and wishes. Immigrants face more challenges, and there is a need to understand their perspectives in addition to those of nonimmigrants. Iranians are one important immigrant group, as their number is limited and their specific needs are less well understood compared to those of the bigger immigrant groups.

**Methods:**

This study used a qualitative explorative research design. Semistructured interviews were conducted between February 2023 and May 2023. The participants were 25 older adults: nonimmigrants residing in cities (n=8, 32%), nonimmigrants residing in rural areas (n=9, 36%), and Iranian immigrants residing in cities (n=8, 32%). Data were analyzed through inductive and deductive content analysis and interpreted through self-determination theory.

**Results:**

Interacting with eHealth services was challenging for some older adults. They perceived several difficulties, with the most obvious ones being related to values and preferences, as some older adults did not value eHealth services (16/25, 64%), had insufficient digital skills (15/25, 60%), and experienced usability issues (15/25, 60%). The first two challenges were more pronounced among immigrants. In contrast, nonimmigrants from cities, being more familiar with the services, shared more usability issues. These identified challenges prevented older adults from satisfying their basic psychological needs of being competent and autonomous users and having a sense of belonging (aspects of self-determination theory), which were the main source of negative emotions. A common negative feeling was confusion (16/25, 64%) among those with limited experience using smart devices and those with poor self-reported digital skills. Conversely, older adults’ interaction with eHealth services generated positive emotions that were connected to the satisfaction of their basic psychological needs. Being interested in using eHealth services was a common feeling among most participants regardless of their background and was connected to satisfying their need for being competent and autonomous. The positive emotions could be supported by applying older adults’ needs to the design of eHealth services (10/25, 40%) and by supporting their digital skills (19/25, 76%).

**Conclusions:**

Some older adults value eHealth services and see their added benefits. However, various challenges limit their use of these services. The analysis of older adults’ needs yielded several practical ideas that could improve the user-friendliness of the services and highlighted the importance of sufficient support services tailored to the cultural needs of specific groups of older adults.

## Introduction

### Background

The growing number of older adults is a significant socioeconomic trend [1]. Over the next 3 decades, the number of older adults—individuals aged ≥65 years—is projected to double [2]. The proportion of older adults in Europe will reach 35% in 2050 [3]. As people age, they may become more susceptible to different diseases, leading to high demand for health care services [4]. However, several barriers limit some older adults from properly managing their health conditions using traditional health care services. These include reduced mobility [5] and residing in rural locations with limited access to physical health care facilities [6]. Furthermore, the number of physical health care facilities is limited and does not match growing needs [7].

To overcome such barriers, digital solutions have been mobilized to deliver eHealth services [8,9], which are defined as “the use of information and communication technologies for health” [10]. eHealth services are transformative solutions in the context of demographic aging, urban normativity, and austerity politics as they aim to enhance health care quality and equality, especially for people in sparsely populated areas [11].

The use of eHealth services can be expected to facilitate communication with health care providers [8] and allow older adults to control their health status at their own pace as they can read their medical records on electronic patient portals [12]. Furthermore, eHealth services could be useful in supporting and managing a healthy diet and lifestyle among older adults [13]. Moreover, eHealth has proven useful in promoting medication adherence as well as effective in preventing disease among older adults [14]. For example, using mobile health can potentially improve health outcomes among older adults with chronic diseases by supporting symptom control [15]. Mobile health refers to the use of mobile phones and other wireless technology for medical purposes, which is a recent technological evolution in eHealth [16].

Despite these benefits, several difficulties hinder some older adults from fully using eHealth services. For example, older adults with limited financial resources might have limited access to smart devices or the internet [17]. Furthermore, some older adults might lack the proper digital skills to use smart devices or e-services [18] and might not be confident in using technology for critical issues such as health [17]. Security issues and privacy concerns are recognized as factors that limit older adults’ willingness to use eHealth services [19,20].

Moreover, these services are often designed without considering older adults’ specific features, such as physical difficulties or low digital skills [21]. Thus, older adults might face several usability issues, such as navigation difficulties [22]. Usability is defined by the International Organization for Standardization 9241-11 standard as the “extent to which a product can be used by specified users to achieve specified goals with effectiveness, efficiency, and satisfaction in a specified context of use” [23]. In addition, immigrant older adults might face more barriers that are related to their backgrounds, such as lower socioeconomic status, low language proficiency, and comparatively lower levels of social inclusion [24]. The perceived difficulties might lead to negative emotions [25,26], lack of interest, and unequal use of eHealth services among older adults, which might increase the risk of inequities and social exclusion [27].

Finland is a very high-income country with advanced health policy programs that ensure citizens’ well-being [28]. To support equal access to public health services, Finnish law supports the provision of e-services to promote the availability, quality, security, and equality of eHealth services for everyone [29]. However, inequalities still exist, especially among vulnerable groups such as older adults and immigrants [17].

To achieve a socially sustainable society in which everyone is treated equally and individuals’ health and functional capacity are supported, it is necessary for people to have a high capability to take responsible action for their health. This can be supported by, for example, tackling the challenges that older adults face in the use of eHealth services and involving older adults from different backgrounds and their specific needs in the design process and delivery strategies of these services [30-32]. This study aimed to understand the perspectives of older adults (aged ≥65 years) from different backgrounds regarding eHealth services to identify their challenges, emotions, and wishes. Achieving this aim could result in ideas for improving the usability of eHealth services for older adults with different backgrounds, generating positive emotions regarding eHealth services among older adults, and encouraging them to use these services.

To achieve the aim of this study, a qualitative analysis of immigrant and nonimmigrant older adults’ experiences with eHealth services was conducted to uncover the challenges they might face, identify the sources of their perceived feelings, and find out their wishes regarding these services. To focus more deeply and consider individuals’ basic psychological needs, this study applied self-determination theory (SDT) to interpret the findings and answer the research questions. SDT is a psychological framework that explains the mechanisms through which individuals develop the ability and motivation to take active roles in various behaviors, such as health care [33].

### Theoretical Framework

SDT suggests 3 basic psychological needs for individuals’ motivation, behavior, and well-being: autonomy, competence, and relatedness [33]. Autonomy refers to self-endorsed reasons and intrinsic motivations, such as enjoyment or perceived benefits, and alignment with one’s core goals for behavioral engagement. Competence refers to a feeling of having the ability and efficacy for development, learning, and expertise. Relatedness is defined as a sense of belonging and having connections with others [31].

SDT can explain support for older adults’ motivation to use eHealth services [34]. Regarding autonomy, eHealth services must provide meaningful choices [35]. According to the concept of competence, older adults must feel able to attain better health outcomes by using eHealth services [36], and the complexity of the services should be appropriate for them [31]. Relatedness refers to fostering meaningful relationships with peers and clinicians, such as being in a short feedback loop with health care providers [37].

SDT has been frequently applied to eHealth adoption [38,39]. For instance, Yuen et al [38] found that individuals’ eHealth adoption is affected by their emotions. The 3 constructs of SDT significantly impact emotions, and emotions are known to act as one of the main sources of human motivation [40]. To elaborate, individuals experience heightened positive emotions when they perceive a sense of control over their circumstances, possess the requisite skills and knowledge to engage proficiently with eHealth services, and feel a strong connection with and care for others [33].

### Research Questions

To achieve the aim of this study, we formulated 3 research questions. The questions explore perspectives of immigrant and nonimmigrant older adults (aged ≥65 years) regarding eHealth services, focusing on their challenges, emotions, and wishes through the lens of SDT. Identifying challenges helps understand barriers preventing older adults from using eHealth services. Recognizing their wishes reveals their needs and expectations. Designing eHealth services based on wishes increases the likelihood of use. Furthermore, by addressing emotions, it is possible to foster positive feelings and encourage greater adoption among older adults. The research questions are as follows: (1) What challenges do older adults face in using eHealth services? (2) What emotions do older adults have regarding eHealth services? (3) What wishes do older adults have regarding eHealth services?

## Methods

### Study Design

This was an explorative qualitative study based on semistructured interviews with 25 older adults aged ≥65 years conducted between February 2023 and May 2023. This study is part of the DigiIN project and was carried out to generate solutions to ensure that social welfare and health care provided via eHealth services are available and accessible to everyone equally and that everyone can use these services. Therefore, we assessed experiences, feelings, and expectations regarding eHealth services among older adults from different backgrounds in Finland.

We selected participants from different backgrounds: nonimmigrant older adults from cities and rural areas and Iranian immigrants residing in cities. The rationale for this selection was to explore and compare the perspectives of different vulnerable groups. Immigrants face more challenges, and there is a need to understand their perspectives in addition to those of nonimmigrants. In addition, compared to the largest immigrant groups in Finland, Iranians are fewer, which limits their access to similar levels of public and peer support to those available for larger groups. Being fewer, their specific needs are likely to be less well understood within the health care system. Therefore, these individuals are one of the key vulnerable groups as per the major demographic change in Western societies [41].

To maintain a balance in participant recruitment based on their living areas, we aimed to recruit Iranian immigrants and nonimmigrants from rural and urban areas. However, this effort was unsuccessful, and we could not recruit immigrant participants from rural areas. We discovered that most immigrants reside in urban areas. This trend is influenced by Finland’s immigration policies and the personal preferences of immigrants.

We used a semistructured interview guide designed for this study by the main researcher and revised with the coauthors (Multimedia Appendix 1). This qualitative study was conducted and reported following the COREQ (Consolidated Criteria for Reporting Qualitative Research) guidelines where applicable [42] (Multimedia Appendix 2).

### Study Setting and Participant Recruitment

We applied the convenience sampling method to recruit the participants. People were eligible if they (1) were aged ≥65 years; (2) were originally from Finland or Iran; and (3) could read and write in Finnish, Swedish, English, or Persian. We excluded people with dementia; those who were too frail or ill; and those who were in active care, such as in hospitals or older adult centers, to reduce possible risks.

We recruited older adults from nonprofit organizations in the Turku and Helsinki regions, including the Turun Seudun Vanhustuki ry (Turku Region Elderly Support Association), Enter Association, Valli Association, and Varissuo Library. These organizations support diversity, inclusion, and the digital empowerment of older adults while fostering intergenerational collaboration. They organize various events and workshops for older adults, giving them access to a broad network within this demographic.

Multiple strategies were used to recruit participants. First, the first author attended events at the aforementioned organizations and directly invited potential participants. Second, the organizations promoted the study to target groups during events, through informal interactions with older adults visiting their facilities, or via social media platforms. Finally, the organizations reached out to potential participants directly to promote the study.

Interested older adults contacted the first author, or the organizations delivered their contact information to her with their permission. The first author then called everyone and inquired about their interest in participating and agreed on the interview time and place. One of the older adults, who was initially interested in participating, did not answer their phone, and 2 refused to participate because of personal issues.

This study included 25 participants, comprising nonimmigrants residing in cities (n=8, 32%), nonimmigrants residing in rural areas (n=9, 36%), and Iranian immigrants residing in cities (n=8, 32%). Most participants were female (17/25, 68%). In total, 56% (14/25) of the participants were aged 65 to 75 years, whereas the remaining 44% (11/25) were aged >75 years. The participants had diverse educational backgrounds: elementary education (5/25, 20%), upper secondary education (1/25, 4%), vocational education (7/25, 28%), and higher education (13/25, 52%). In addition, 6 participants had medical education: 5 (83%) were nonimmigrants, and 1 (17%) had an immigrant background.

### Collecting Data

To collect data, we used the designed semistructured interview guide. We collected information about the participants’ backgrounds, including their age, gender, and educational level. Moreover, we asked questions about participants’ experiences with eHealth services, including whether they perceived any benefits or reasons for not benefiting. We also inquired about their emotions while interacting with these services and their suggestions for improving eHealth services.

We piloted the designed interview guide with 1 immigrant and 1 nonimmigrant older adult. As no major revisions were necessary, we included the pilot interviews in the study with participants’ consent. All interviews with immigrant and nonimmigrant older adults from rural areas (17/25, 68%) were conducted over the phone. A total of 50% (4/8) of the interviews with nonimmigrant older adults in the cities were conducted on the web via Zoom (Zoom Video Communications), and the other 50% (4/8) were conducted face-to-face in the Turun Seudun Vanhustuki ry premises.

All interviews were conducted by the first author between February 2023 and May 2023. Participants were interviewed in their preferred languages: English, Swedish, Finnish, or Persian. The interviews were digitally audio recorded with the participants’ permission. After each interview, the audio files were transcribed into text files and then deleted. All interviews were translated from Finnish, Swedish, and Persian into English for further analysis. We used transcription and translation services for transcribing and translating the recorded files, resulting in a total of 318 pages. The transcribed files and findings of the study were not revised by the participants.

The average length of the interviews was 35 (SD 8.54) minutes. We continued with the interviews until we reached the saturation point—when we had collected sufficient data to answer the research question or objective and data collection did not result in new analytic codes or themes [43].

### Data Analysis

The data were analyzed using a combination of both inductive and deductive content analysis to understand the information domains better [44]. The combination of inductive and deductive content analysis is widely used across various research areas [17,45] and is considered the most realistic approach for incorporating theory and literature into the analysis [44]. To analyze the data, 3 iterative coding steps—reduction, grouping, and abstraction—proposed by Elo and Kyngäs [44] were applied.

The unit of analysis comprised a collection of ideas in which participants detailed the challenges they faced with eHealth services along with their emotions and wishes regarding these services. During the data reduction process, all researchers thoroughly read through the transcripts and used open coding to accurately summarize the set of ideas. All researchers had previous experience in qualitative research. The interview transcripts were then coded collaboratively by all the researchers. The codes were then grouped based on similarity and given descriptive names. These subcategories were reviewed by the main researcher, who further organized them into higher-level categories: challenges (n=7 main themes), emotions (n=2 main themes), and wishes (n=2 main themes). All subcategories were then linked to the 3 basic psychological needs based on SDT where applicable [33]. The ATLAS.ti software (version 24; ATLAS.ti Scientific Software Development GmbH) facilitated the analysis of the data.

### Ethical Considerations

The Aalto University ethics review board granted the ethical permission to conduct this study (reference D/1220/03.04/2022). Participation was voluntary, and participants were informed of their right to withdraw from the study at any time without providing a reason. No previous relationships existed between the researchers of the study and the participants.

Before the interviews, we provided participants with oral information about the study, its aims, ethical processing, data collection, preservation of data, and reporting of results. To ensure that the participants were informed, we provided them with a written information sheet. Written informed consent was sought from all participants. Participants received the information in their preferred languages: English, Swedish, Finnish, or Persian.

We anonymized the data to protect the interviewees’ information and stored them on a secure local server with strong password protection. All participants were offered a small thank-you gift. The gift was a water bottle provided by the DigiIN project valued at <€20 (US $20.90).

## Results

### Challenges of Using eHealth Services

#### Overview

The participants had different experiences with eHealth services. Some of those with medical education shared that they had no major issues using eHealth services for personal purposes, but the rest considered eHealth services challenging and reported several problems, explained in the following sections and summarized in Table 1. In the participant IDs, *C* refers to participants from cities, *R* refers to participants from rural areas, and *I* refers to Iranian immigrants.

**Table 1:** Immigrant and nonimmigrant older adults’ perceived challenges regarding eHealth services in Finland and the links to the basic psychological needs introduced in self-determination theory (SDT).

Challenges		Link to SDT	Nonimmigrants in cities	Nonimmigrants in rural areas	Iranian immigrants
**Barriers related to values and preferences**					
	Valuing in-person visits	Lack of relatedness	✓	✓	✓
	Lack of interest	—^a^	✓	✓	✓
	Perception of not needing eHealth services	—	✓	✓	✓
	Preference for delegating health care responsibilities	—			✓
**Usability issues**					
	Complexity	Lack of competence	✓	✓	✓
	Technical terminology	Lack of competence	✓	✓	
	Lack of services in users’ native languages	Lack of autonomy and lack of competence			✓
	Log-in difficulties	Lack of competence		✓	✓
	Lack of functionality for leaving comments on or correcting errors in physicians’ notes on the patient portal	Lack of autonomy and lack of relatedness	✓		
	Lack of possibility to contact health care providers directly through the patient portals	Lack of relatedness	✓		
**Lack of digital skills**					
	Inadequate digital skills for using eHealth services	Lack of competence	✓	✓	✓
	Inadequate digital skills for using smart devices	Lack of competence			✓
	Lack of proper navigation skills	Lack of competence	✓	✓	✓
	Difficulty following updates	Lack of competence	✓	✓	
**Lack of support**					
	Lack of family support	Lack of competence	✓	✓	
	Lack of support close to their living area	Lack of competence		✓	
	Lack of proper support and meeting their needs	Lack of competence	✓		
	Lack of support in their native languages	Lack of competence			✓
**Lack of self-efficacy**					
	Fear of making a serious mistake	Lack of competence	✓	✓	✓
	Doubting their abilities to manage unwanted situations	Lack of competence	✓	✓	✓
	Doubting their local language proficiency	Lack of competence			✓
**Health-related challenges**					
	Vision and memory issues	Lack of competence	✓	✓	✓
	Shaky hands	Lack of competence			✓
**Lack of access to digital resources**					
	Lack of strong e-ID	—		✓	✓
	Lack of access to smart devices or the internet	—			✓
	Lack of familiarity with eHealth services and their value	—		✓	✓

^a^Not applicable.

#### Barriers Related to Values and Preferences

Some participants (16/25, 64%), even those with successful experiences using eHealth services, reported a preference for face-to-face visits or using eHealth services along with in-person visits, especially for important issues. The participants found it challenging to interact with health care providers and express their issues via web-based health platforms. Therefore, face-to-face visits were assumed to be more effective and reliable in reducing the risk of misdiagnosis. The communication-related challenges were stronger among immigrants because they missed the possibility of using translation services or body language during online visits:

When doctors visit, I feel they understand health problems better. Explaining health issues over the phone or via text is not enough; I might miss sharing some of the symptoms, or there might be misunderstandings. I don’t want to end up eating the wrong medicines.

R2

Some participants reported that the use of eHealth services could decrease their human-based interactions in general, so they preferred to avoid it. They referred to their loneliness and noted that visiting health care providers, often due to their illness, was their main form of face-to-face interaction:

I am sick and can’t go out alone. Going to hospitals is my only chance to be outdoors and meet people. If I use eHealth, I’ll lose this chance.

C4

Some participants did not see the added value of eHealth services and, therefore, lacked interest in using them. They mentioned that these services did not match their needs or that identifying a suitable e-service required more time and energy than they could manage. They also noted that not all medical needs could be treated on the web, at least not for their generation.

Furthermore, some participants felt that eHealth services were designed for sick people. Given their self-identification as healthy individuals, they perceived such services as unnecessary and preferred not to use them. In addition, some immigrants noted their preferences for delegating their health care responsibilities to family members as they assumed themselves to be old and incapable of managing health issues, especially in their nonnative languages. In addition, they believed that, just as they had cared for their parents, their children should take care of them. Therefore, they did not feel the need to use eHealth services:

I don’t know where to go or what to do. I don’t even know the language very well. It is better if my kids handle things. When my mother was old and sick, I was taking care of her, and my kids are doing the same for me.

I4

#### Usability Issues

Some participants (15/25, 60%), especially nonimmigrants residing in cities, raised several usability issues. The primary usability concern was the intricate nature of these services and their improper interface design, which resulted in the overload of information, hidden information, and navigation difficulties. Participants reported several navigation challenges, including difficulty distinguishing between clickable and nonclickable elements in some health-related web pages:

On some pages, it’s hard to know where to click, where not to click, or even when clicking is an option.

R1

The usability issues reported mainly concerned My Kanta, the Finnish national patient portal. Regarding complexity, the participants noted that the main page of My Kanta is overloaded with extra information, which makes it hard to find relevant information. They also added that clicking on certain elements on My Kanta often led to new pages overloaded with excessive details, which they found overwhelming:

I clicked on a link it opened another window with much information. Made it even more difficult to find what I need.

R3

Furthermore, some participants raised concerns about navigating back to the home page, stating that, after several clicks, they found it challenging to return to the home page quickly. Another important usability issue was related to the language. Immigrants said that their effective use of eHealth services was diminished by the lack of services in their native languages, whereas nonimmigrants considered medical or technical terminologies to be barriers to their efficient use.

Some participants, especially those with memory issues, reported that the log-in process to My Kanta was difficult and time-consuming. They also expressed frustration with being required to log in again due to session timeouts or opening another window.

Some nonimmigrants in cities also expressed discontent caused by the lack of functionality for leaving comments on or rectifying errors in physicians’ notes on patient portals. This problem was said to limit users’ autonomy in managing the trustworthiness of their information. In addition, participants reported feeling disconnected from health care providers. The same participants also shared that the lack of possibility to contact health care providers directly through My Kanta further contributed to this sense of disconnection:

I had an accident a few weeks ago and went to the hospital. A couple of days after my visit, I checked My Kanta to read the doctors’ notes. There was false information, and I couldn’t get rid of it or leave a comment on it. The note said that I had fainted when I went to the hospital, but I hadn’t. With this wrong information, what’s the point of using My Kanta then?

C6

#### Lack of Digital Skills

Some participants (15/25, 60%) reported challenges due to their lack of the adequate digital skills needed to use smart devices and the internet for critical issues such as health. The participants reported using smart devices and the internet to meet their daily needs, such as contacting their networks or using social media, but not for serious matters. They felt that using eHealth services required a high level of digital skills compared to using other e-services:

I have a smartphone, I call people, and I am active on Facebook, but I don’t use digital health services. You must be good enough with computers to use them for health issues. I am not.

R5

Furthermore, some participants, especially immigrants, reported that their insufficient digital skills caused them difficulties navigating eHealth platforms. This perceived difficulty led to issues with finding information:

I can’t find the information I need on My Kanta; I don’t have skills for that.

I3

Some nonimmigrant participants expressed that their lack of adequate digital skills made it challenging to follow updates in eHealth services or health apps. They considered themselves slow adopters of technology. Moreover, 25% (2/8) of the immigrants reported having difficulties dealing with smart devices in general due to their lack of experience with technology-based tools.

#### Lack of Support

The lack of support was a prevalent concern among some participants (14/25, 56%). The lack of support, they said, could create the impression that these services are not intended for older adults, especially for those who are not capable of using smart devices. Some nonimmigrants cited a lack of family support. They either lacked a family member to turn to with their queries or hesitated to seek assistance believing that their family members could not satisfy their learning needs. Furthermore, nonimmigrants in cities mentioned that either they were unfamiliar with the available support or the available support did not satisfy their needs. Nonimmigrants in rural areas reported a lack of support close to their living areas, and immigrants noted a lack of support in their native languages.

#### Lack of Self-Efficacy

A total of 44% (11/25) of the participants reported a fear of making serious mistakes while using eHealth services. They were afraid that something irreversible might happen, leading to unwanted and unpleasant results with long-term consequences, such as the nonrenewal of prescriptions or the deletion of important information. They were worried and doubted their capabilities to manage the situation:

I use computers for writing emails or watching videos, but I never dare to use eHealth services alone. I don’t want to make any mistakes or cause any problems. Not for me not for doctors.

I2

Furthermore, some immigrants, even those with a good understanding of the Finnish language, mentioned a lack of trust in their Finnish-language proficiency. This lack of trust prevented them from using the eHealth service in Finnish.

#### Health-Related Challenges

Health-related challenges such as memory or vision problems were common among 36% (9/25) of the participants and caused difficulties remembering log-in passwords, navigating, or using e-services on mobile phones with small screens. Some participants also mentioned that shaky hands caused difficulty holding phones or tablets or typing text.

#### Lack of Access to Digital Resources

Some participants shared basic challenges that prevented them from using eHealth services. For example, 25% (2/8) of the immigrants reported a lack of access to smart devices (eg, computers or smartphones) and the internet. In total, 62% (5/8) of the immigrants noted their lack of familiarity with the available eHealth services. However, the lack of general familiarity was only an issue among immigrant older adults. Participants from all groups mentioned a lack of familiarity with the functionalities and benefits of eHealth services:

I know about this Kanta; I have heard from my children. But I don’t know what exactly it has for me.

R6

Moreover, 25% (2/8) of the immigrants and 11% (1/9) of the nonimmigrants in rural areas shared that they did not have a strong e-ID code, which is a prerequisite for using public e-services in Finland. Therefore, they could not use some e-services such as My Kanta.

### Emotional Experiences Using eHealth Services

#### Overview

Participants’ experiences with eHealth services generated positive and negative emotions, which are analyzed and summarized in Tables 2 and 3.

**Table 2:** Immigrant and nonimmigrant older adults’ positive emotions regarding eHealth services in Finland and the links to the basic psychological needs introduced in self-determination theory (SDT).

Positive emotion	Definition	Link to SDT	Nonimmigrants in cities	Nonimmigrants in rural areas	Iranian immigrants
Trust	Feeling that eHealth services are safe, secure, and reliable	—^a^	✓	✓	✓
Interest	Willingness to learn how to use eHealth services as people understand their usefulness or consider them easy to use	Competence	✓	✓	✓
Interest	Willingness to use eHealth services as they offer meaningful choices to satisfy patients’ needs and people feel that they have enough skills to use these services	Autonomy and competence	✓	✓	✓
Being empowered	Having more control over their health situation, making independent decisions, and being more involved in their health care management	Autonomy and competence	✓	✓	✓
Being less worried	Being less worried about their health condition	—	✓	✓	✓
Being connected	Being connected to health care providers	Relatedness	✓	✓	✓
Hope	Reduction in migration rate from rural areas for health care purposes and hoping to save their community	—		✓	
Peace of mind	Access to health care services in rural areas; freedom to live in their favorite places	Autonomy		✓	
Equality	Equal access to health care services in the country	—		✓	

^a^Not applicable.

**Table 3:** Immigrant and nonimmigrant older adults’ negative emotions regarding eHealth services in Finland and the links to the basic psychological needs introduced in self-determination theory (SDT).

Negative emotion	Definition	Link to SDT	Nonimmigrants in cities	Nonimmigrants in rural areas	Iranian immigrants
Confusion	Dealing with the complicated interface design of eHealth services was confusing	Lack of competence	✓	✓	✓
Lack of interest	Not willing to use eHealth services as they are complex and the use of such services could limit in-person interactions with health care providers	Lack of competence and lack of relatedness	✓	✓	✓
Lack of interest	Not willing to learn how to use eHealth services for different reasons, such as being old and incapable of learning	Lack of competence	✓	✓	✓
Frustration	Feeling frustrated with learning and using complex eHealth services	Lack of competence	✓	✓	✓
Stress	Feeling stress when using complex eHealth services	Lack of competence	✓	✓	
Dependency and shame	Feeling shame of being dependent on others and asking questions even regarding simple issues	Lack of autonomy and lack of competence	✓	✓	✓
Use by force	Feelings of being forced to use eHealth services	Lack of autonomy	✓	✓	
Alienation	Feelings of being excluded due to lack of services in their native languages	Lack of relatedness			✓
Disconnection	Lack of direct connection with health care providers via My Kanta to discuss recorded notes	Lack of relatedness	✓	✓	

#### Positive Emotions

Our participants shared several positive emotions. Most (19/25, 76%) expressed a prominent level of trust in eHealth services in Finland and the security of their online medical records. This was attributed to trust in the authorities and the Finnish government:

I believe that the Finnish government is taking care of the security of our health data.

C2

Some participants (13/25, 52%) reported an interest in learning about and using eHealth services. They found the services easy to understand and use. The nonimmigrant participants attributed eHealth services’ perceived ease of use to their previous experience with computers and the internet. In addition, 47% (8/17) of the nonimmigrants found the services useful and appreciated the convenience and time savings they offered. They believed that these services provided meaningful options to satisfy different health-related needs, for example, booking appointments or adhering to the treatment process:

I like these digital health services; they help me a lot. I have an app on my phone that reminds me to take my medications. Without the app, I might miss to take my pills. Or...this hospital app that I use [Terveystalo], I text doctors. The same app helps me book appointments.

C2

The immigrant participants also shared their interest in learning about and using the services but without giving any specific reasons. However, some of them expressed interest in learning about and using these services after we provided them with information on their usefulness.

Some participants (9/25, 36%) reported feeling empowered by using eHealth services for different reasons. First, they said that having access to their medical records enabled them to control their health situation, such as renewing prescriptions and making informed decisions such as booking or canceling an appointment:

By using My Kanta, I control my medicines and renew my prescriptions if I need them. I read my lab results or doctors’ notes carefully, then I decide to book or cancel an appointment. And sometimes, I prepare some questions to ask in my visits based on my medical records.

R1

Second, some participants described feeling empowered by the possibility of contacting health care providers at any time through the chat option. This functionality allowed them to be more actively involved in managing their health care.

Furthermore, 36% (9/25) of the participants found that access to their medical records helped them enhance their understanding of their health status and reduced their worries. Some participants, especially those living far from physical health care facilities, reported positive feelings about being connected to health care providers via chat or phone calls. Nonimmigrants in rural areas shared 3 positive feelings: equality in terms of using health care services in the country, hope that the use of eHealth services could save their community by reducing the migration rate from rural to urban areas for health care purposes, and peace of mind to live alone in rural areas due to access to eHealth services from their home. The availability of eHealth services was said to offer the autonomy to choose preferred living arrangements:

I live in an archipelago alone, far from hospitals. I have appointments with doctors online when the issue is not urgent. I don’t need to travel to cities for that reason. People used to move to cities when they were old or sick, but nowadays it is possible to do things online and live in archipelagos, at home.

R4

#### Negative Emotions

Our participants reported several negative emotions regarding eHealth services, with confusion being the most frequently mentioned. A total of 64% (16/25) of the participants, especially those with limited experience using smart devices and those with poor self-reported digital skills, reported that the interface design of eHealth services was complex and confused them, at least in their first experience:

In my first experiences with My Kanta, I found it very complex and confusing. I had no idea where the information was or what to do. It is still confusing but not like the first time.

C6

The complexity of the services made some participants (7/25, 28%) feel that these services were not designed for people of their age and could not satisfy their needs. Thus, they did not like to interact with such services*.* In addition, they were afraid of missing in-person interactions with health care providers. Moreover, 28% (7/25) of the participants were not interested in learning new skills as they assumed themselves to be very old and incapable of learning how to use eHealth services. Even those who aimed to learn about eHealth services found it frustrating and noted that the complexity of the services made the learning process more challenging:

I am too old and don’t want to learn new things; even if I want to, I can’t. It is hard for me to memorize and remember. I just want you to make things that I know how to use easier.

I5

In total, 28% (7/25) of the participants, those with experience using eHealth services, experienced stress or were irritated when interacting with such complex services. Furthermore, some participants (6/25, 24%) reported that the use of eHealth services made them dependent on others and feel ashamed. As they did not have enough digital skills or experience with computers or e-services, they found the services complex and had to ask for help constantly.

Moreover, 35% (6/17) of the nonimmigrants felt that engaging with eHealth services was somehow obligatory. Despite their initial reluctance, they perceived an external force to adopt these services to remain connected with their community, avoid falling behind youth, and keep pace with developments in health technology:

When my kids told me about these digital health services, I found them very difficult to learn, but I said I had to learn them. If I don’t learn now, I will be an outsider soon. I think everything will be digitalized soon.

C5

In addition, most immigrants (5/8, 62%) reported feelings of being excluded as the available eHealth services were not in their native languages. Nonimmigrant participants expressed a different concern regarding being disconnected. They mentioned that the lack of possibility to contact health care providers directly through My Kanta to discuss recorded notes caused feelings of disconnection:

I feel like these services aren’t meant for me. If they wanted me to use these eHealth services, they would make them available in my language.

I7

### Wishes Regarding eHealth Services

#### Overview

Participants’ interactions with eHealth services and their perceived challenges shaped their wishes regarding these services. The identified wishes are explained in the following sections and summarized in Table 4.

**Table 4:** Immigrant and nonimmigrant older adults’ wishes regarding eHealth services in Finland and the links to the basic psychological needs introduced in self-determination theory (SDT).

Wish			Definition		Link to SDT		Nonimmigrants in cities		Nonimmigrants in rural areas		Iranian immigrants
**Wishes for all kinds of eHealth services**											
	User-friendly design	Simple interface		Competence		✓		✓		✓	
	Support services	Improve digital skills and efficacy		Competence		✓		✓		✓	
	Unified platform	A single local system		Competence		✓		✓			
	Language	eHealth services without technical terminology		Competence		✓		✓			
	Language	eHealth services in users’ native languages		Competence and autonomy						✓	
	Facilitate log-in	Log in to eHealth services without an e-ID		Competence				✓		✓	
	Fewer updates	Zero to few updates		Competence		✓		✓			
**Wishes only for My Kanta**											
	Speed up access	Upload medical records on My Kanta immediately		—^a^		✓		✓			
	Notification	Sending notifications to users’ mobile phones when new records are uploaded		—		✓					
	Edit or comment	Possibility to edit or comment on physicians’ notes on My Kanta		Autonomy		✓		✓			
	Direct contact	Discuss queries on the recorded health data with health care providers via My Kanta directly		Relatedness		✓		✓			

^a^Not applicable.

#### Identified Wishes

In Finland, several similar eHealth platforms offer the same services, leading to confusion among users. To address this issue, 40% (10/25) of the participants suggested creating a unified platform that consolidates these services:

It is confusing when different sites are giving the same services. It takes time and energy to learn how to use them. It’s good if, in one place, you can do very many different things.

R4

This single platform was expected to have a user-friendly design for all user groups. Our participants shared several suggestions, such as having only main headlines with big font sizes on the home page; using icons alongside each heading to facilitate information seeking; and minimizing the use of large images on the home page as it might cause navigation difficulties, especially on mobile devices. In addition, some participants emphasized incorporating color into the design. The coloring system was suggested as a potential aid for navigation, especially for people with vision impairments.

Several suggestions were given specifically for My Kanta. These included adding the possibility of listening to the medical records or physicians’ notes, the possibility of navigating back to the home page by clicking on the large Kanta icon in the corner of the web page, sharing medical records without any delay, sending notifications to users’ mobile phones for new or unchecked records, and the availability of a hotline or chat option as a channel to contact health care providers directly to discuss queries or share worriers about the recorded medical data. These participants wished to have the possibility to edit or comment on physicians’ notes, too:

Sometimes when I read doctors’ notes, I find them hard to understand or I disagree. I wish there was a direct channel to call or text the same doctor who has written the notes and discuss.

C3

Furthermore, due to the importance of completely understanding health information, immigrants wished to have at least critical health information in their native languages, and nonimmigrants suggested that health information should be free of medical terminology. In addition, some participants shared the idea of having the possibility to log in without an e-ID. In Finland, to increase security, an e-ID is necessary to log in to public e-services; however, some participants, especially immigrants, found this challenging. Moreover, some participants wished for services without any updates or that, in the case of changes, there should be announcements and proper support. Tailored support in individuals’ native languages and close to their living areas was requested by most participants (19/25, 76%).

## Discussion

### Principal Findings and Comparison With Prior Work

This study used a qualitative exploratory approach to investigate the perspectives of immigrant and nonimmigrant older adults on eHealth services, focusing on their perceived challenges, emotions, and wishes. In addition, it highlighted the role of the basic psychological needs of competence, autonomy, and relatedness, as outlined in SDT, in shaping these perspectives. To support the use of eHealth services by older adults regardless of their background, our findings outline several practical recommendations for developing user-friendly eHealth services and digital supports.

We found that the most common barriers among immigrant and nonimmigrant participants were their values and preferences, inadequate digital skills, and usability issues. Immigrants faced more challenges related to personal values and preferences and inadequate digital skills, whereas nonimmigrants complained that their eHealth use was mainly diminished by usability issues.

We found that barriers related to values and preferences hindered older adults’ use of eHealth services for different reasons. First, some participants were reluctant to use eHealth services as they did not understand their added value or highly valued in-person visits to overcome web-based communication challenges. Communication issues varied among participants by background. Although a common problem was the fear of losing human-based interactions, the difficulty in using translation services or body language hindered immigrants without proficiency in the local language from using eHealth services. These findings align with the findings of Wang et al [46]. We showed that the communication-related barriers prevented older adults from satisfying their need for a sense of belonging, reflected in the relatedness aspect of SDT.

Second, participants preferred to use only physical health care facilities or eHealth services aligned with existing in-person services to avoid misdiagnoses, which is in line with previous studies [47,48]. Third, our findings add to the body of knowledge regarding the fact that some Iranian older adults intentionally preferred to delegate their health care responsibilities to their family members and were reluctant to be involved in their health care. Therefore, they did not feel a need to learn about eHealth services. They justified their behavior with their lack of familiarity with the local health care system, language issues, and their own culture. This finding suggests that, even if issues related to language barriers and familiarity with the local health care system are resolved, some Iranian older adults may still prefer to rely on caregivers such as their children. This preference is rooted in their cultural values, which emphasize that older adults should be cared for by younger family members.

The importance of culture has been highlighted previously. Previous studies have considered culture an enabler of learning and using eHealth technologies among some older adults, especially immigrants. The literature shows that older adults’ use or nonuse of eHealth services relates to their technology-related cultural perceptions and the contextual factors of their environment and lifestyle [30,49,50]. DeLange Martinez et al [51] identified ethnicity and culture as significant predictors of technology use among Asian immigrant older adults.

Another important group of challenges related to digital skills. Participants’ challenges with inadequate digital skills manifested in various ways. Most nonimmigrants and some immigrants felt that their digital skills did not ensure their proficiency in using eHealth services; however, they could use smart devices and search the internet for noncritical topics. In contrast, some immigrants faced greater challenges; they had not been taught the basic digital skills required to use smart devices and the internet in general. These findings are in line with those of previous studies [17,18]. We found that all types of difficulties in terms of digital skills prevented older adults from satisfying their need for a feeling of being competent users of eHealth services (competence aspect of SDT).

Moreover, we identified several usability issues raised mainly by nonimmigrant participants residing in cities. This group of participants had more experience with eHealth services; therefore, they had more knowledge regarding usability issues. A commonly identified usability issue among participants regardless of their immigrant or nonimmigrant status was the complexity of eHealth services and their intricate interfaces. This challenge has been highlighted in previous research [12,17,52,53]. However, the perceived complexity was dependent on other background characteristics; for instance, participants with medical backgrounds found these services less complicated than other participants.

The perceived complexity of eHealth services coupled with inadequate digital skills prevented older adults from satisfying their basic psychological needs outlined by SDT and generated feelings of confusion, stress, and frustration when interacting with eHealth services. Similarly to the study by Chen et al [24], we showed that failure to satisfy the psychological need for being competent users of eHealth services (competence aspect of SDT) led to negative feelings of frustration and anxiety among older adults. Moreover, we add to the literature that the 2 aforementioned challenges of perceived complexity and inadequate digital skills led some participants to repeatedly seek assistance when using the services. On the basis of SDT, these challenges hindered older adults from satisfying their basic psychological need for feelings of being autonomous and competent users of eHealth services (autonomy and competence aspects of SDT), resulting in feelings of shame and reliance on others.

The second most common usability issue—language-related challenges—varied among immigrant and nonimmigrant older adults. Medical terminology use was challenging for nonimmigrants, whereas immigrants faced a lack of eHealth services in their native languages, similar to the findings of Kaihlanen et al [17]. Lack of services in immigrants’ native languages leads to the experience of alienation and exclusion (ie, hindering them from meeting their basic psychological need for a sense of belonging [relatedness aspect of SDT]). This discovery aligns with the findings of Safarov [54]. Our findings contribute to the literature by revealing that other usability issues prevented the satisfaction of the basic psychological need for a sense of belonging among nonimmigrant participants. Nonimmigrants felt disconnected due to the lack of functionality for leaving comments correcting errors in physicians’ notes on My Kanta or contacting health care providers directly through My Kanta to discuss their medical records.

Our findings highlight other differences between immigrant and nonimmigrant groups. We found that, despite the perceived challenges and initial reluctance to use eHealth services, some nonimmigrant older adults felt obligated to use these services as a means of staying connected with their community and keeping pace with younger generations. The sense of obligation to adopt innovations may be influenced by Finnish culture or social norms, although we lack concrete evidence to substantiate this claim. This feeling prevented some nonimmigrant older adults from satisfying their psychological need for the feeling of having freedom of choice in using eHealth services (autonomy aspect of SDT). Moreover, Kaihlanen et al [17] showed that not possessing an e-ID was a barrier for immigrants to access eHealth services. Our study added to their findings in that some nonimmigrants residing in rural areas had the same issue.

In addition to the perceived challenges and negative emotions, older adults’ interactions with eHealth services generated several positive emotions. Fostering them could support older adults’ motivations to use eHealth services. We found that some older adults were interested in learning about eHealth services and using them. However, only nonimmigrants described reasons for their interest. First, they perceived such services as easy to interact with to manage some of their health care needs. This perception signals their basic psychological need for a feeling of being competent users of eHealth services (competence aspect of SDT) being satisfied. Second, nonimmigrants justified their interest as eHealth services giving them access to different meaningful options to satisfy their health care needs independently, which was linked in the analysis to the satisfaction of their psychological need for autonomy. These findings regarding reasons supporting nonimmigrants’ interest in eHealth services confirm those of previous studies [55,56].

Moreover, we found that nonimmigrants residing in rural areas expressed more positive feelings compared to others, including equality, hope, and peace of mind, which in the analysis was considered to stem from the satisfaction of their basic psychological need for autonomy in selecting their living places due to the possibility of using eHealth services from rural areas. In addition, satisfaction with being able to contact health care providers (relatedness aspect of SDT) led to the positive feeling of being connected, for example, through the possibility of using chat or phone call options to contact health care professionals for nonemergencies.

Overall, the conducted interviews with older adults yielded several insights for supporting their use of eHealth services that are tied to supporting their basic psychological needs. The first suggestion was related to the design of the services. The participants complained about various available eHealth platforms offering similar services and mentioned being confused. Thus, they suggested that a concrete improvement could be to develop a unified eHealth platform that consolidates multiple services. This suggestion has been made previously [56,57]. However, instead of developing a completely new platform, we recommend that this could be realized through complementing existing patient portals with missing features beyond those available in their current versions. Our key recommendations are to include within such portals options for direct web-based connections with health care providers to discuss older adults’ queries about their medical records and the possibility of commenting on their medical records. Such options could satisfy the psychological need to feel connected (relatedness aspect of SDT) among older adults. To satisfy the need for autonomy (as per SDT), we suggest developing the services in multiple languages and using plain terminology. In addition, the platform should have a user-friendly interface design by incorporating visualization tools such as icons alongside each heading, using coloring systems that facilitate navigation, simplifying log-in procedures, and minimizing the frequency of changes to the user interfaces. Adding these features could support satisfying the need to feel competent among older adults.

The second recommendation was to support older adults’ digital skills considering their backgrounds. The importance of digital support has been well documented in previous studies [24,30]. Our findings confirm this need, highlighting that existing support services remain insufficient or unsuitable for the diverse needs of older adults, especially immigrants. Our findings confirm that digital support services should be provided in multiple languages considering the cultural differences and needs of different groups of older adults. Similarly, Anand et al [58] highlighted the importance of considering the country of origin and cultural factors in educating older adults. As a practical solution to promote culturally oriented digital support for immigrants, we recommend that translators assigned by the local health care system to assist immigrant older adults during health care visits also be trained to provide digital support services tailored to their specific needs and culture. However, before introducing digital support services, we suggest raising awareness among older adults, particularly immigrants, about the benefits of independently managing health issues via eHealth services. This foundational information can be disseminated through multiple channels. For example, the government agencies responsible for administering national social security programs with contact to all residents could serve as an effective channel. Moreover, to address the identified issue of older adults’ reluctance to use eHealth services, which they view as replacing personal interactions that serve a vital social function for lonely individuals, we suggest that society prioritize combating loneliness as a significant and separate social challenge.

### Limitations

This study’s major limitation lies in its sample selection as it exclusively included immigrants from Iran, who are ranked 13th on the list of the most populous groups of immigrants in Finland [57]. Several reasons supported this selection. First, the first author was proficient in Persian, enabling effective communication with immigrant participants. Second, this group of immigrants has never been the focus of similar studies in Finland; therefore, their perspectives and needs regarding the Finnish health care system were unknown. This limitation impacts the generalizability of the findings to other immigrant groups in Finland and elsewhere even if we find it plausible that the observed challenges are not limited to Iranians in Finland. For the future, we recommend a larger study with participants from various countries.

Another limitation is the lack of focus on a specific eHealth service. Instead, we allowed participants to raise their most important challenges concerning any type of eHealth service, which allowed us to survey the eHealth landscape more broadly from the user perspective. In addition, this study only compared results based on participants’ background information regarding their rural or urban residency and whether they were immigrants. To gain a deep understanding of the background information, we recommend conducting a study that compares the use of eHealth services based on other background information, such as educational level or occupation. It is worth noting that some participants had a medical background. Furthermore, this study collected data solely through interviews and self-reports without conducting usability tests. We recommend a similar study with a follow-up usability test.

### Conclusions

The results of 25 interviews with immigrant and nonimmigrant older adults indicate that, while the use of eHealth services is easy for minority groups with medical backgrounds, others face several difficulties. Personal values and preferences, insufficient digital skills, and usability issues were common challenges. The first 2 challenges were more obvious among immigrant older adults, whereas nonimmigrants shared more usability issues due to their familiarity with the available eHealth services. The perceived challenges prevented older adults from satisfying their basic psychological needs for competence, autonomy, and relatedness and generated negative emotions. The Iranian immigrant participants shared more negative feelings regarding eHealth services as they faced more challenges.

To support older adults in the pursuit of well-being via eHealth services, users’ basic psychological needs and their wishes regarding eHealth services should be considered from the design stage to the development and implementation processes. To meet this goal, developing a unified eHealth platform with a user-friendly design that consolidates multiple services is of crucial importance to mitigate perceived complexity and the need for advanced digital skills to use such a service because most older adults are concerned that their digital skills are not enough to use complex eHealth services. In addition, a tailored digital support service considering the cultural diversity of users should be available and accessible in multiple languages for older adults. However, before offering digital support, it is essential to promote the importance of managing health issues independently, particularly for those who prefer to delegate their health care responsibilities to their caregivers. These actions could foster positive feelings and support older adults’ motivation to use eHealth services.

## Appendix

Multimedia Appendix 1 Interview guide.

Multimedia Appendix 2 COREQ (Consolidated Criteria for Reporting Qualitative Research) checklist.

## Abbreviations

COREQ: Consolidated Criteria for Reporting Qualitative Research
SDT: self-determination theory

## Data Availability

The datasets generated or analyzed during this study are not publicly available and cannot be provided upon request due to security and privacy concerns.
