# Supplementary material for: Experiences and Expectations of Immigrant and Nonimmigrant Older Adults Regarding eHealth Services: Qualitative Interview Study
Source: J Med Internet Res. 2025 Mar 14;27:e64249. doi: 10.2196/64249 (PMC11953591; doi:10.2196/64249)
Supplement: Multimedia Appendix 1 [file jmir_v27i1e64249_app1.docx]

**The Interview Guide.**

1. Warm-up: Background questions:
   1. Age
   2. Gender
   3. How many years in total have you had an education?
   4. Do you have medical training? If yes what was your field of study?
   5. Current or previous occupation [in a case if they are retired]
2. Do you have access to smart devices, like mobile phones, computers, or tablets, connected to the internet? If yes, which one?
3. Do you have experience of using smart devices?
   1. If yes, could you share more information on what smart devices have you used and why?
4. Have you used any eHealth services, such as My Kanta or mobile health applications?
   1. If you have experience, which eHealth services have you used?
   2. If you do not have experience, why do you think you did not use eHealth services?
5. Could you please share your experiences of using eHealth services?
   1. In your opinion, has the use of eHealth services given you help, support or other benefits? What kind of? (If no: Why?)
   2. What thoughts and feelings have emerged for you? Could you give some examples?
   3. How do you think eHealth services have functioned? What has functioned well? What has not functioned well? Can you describe it?
6. Could you please share your opinion on how the use of eHealth services could be supported?
   1. How do you think eHealth services should be developed? Or based on your experiences, what suggestions do you have for improving eHealth services?
   2. In your opinion, what kind of support do you feel could be helpful? Could you give some examples? Or in your opinion, what can encourage older adults to use eHealth services?
